# Drug Release by Direct Jump from Poly(ethylene-glycol-b-ε-caprolactone) Nano-Vector to Cell Membrane

**DOI:** 10.3390/molecules21121643

**Published:** 2016-11-30

**Authors:** Ugo Till, Laure Gibot, Anne-Françoise Mingotaud, Jérôme Ehrhart, Luc Wasungu, Christophe Mingotaud, Jean-Pierre Souchard, Alix Poinso, Marie-Pierre Rols, Frédéric Violleau, Patricia Vicendo

**Affiliations:** 1 Université de Toulouse, UPS/CNRS, IMRCP, 118 Rte de Narbonne, 31062 Toulouse, France; till@chimie.ups-tlse.fr (U.T.); afmingo@chimie.ups-tlse.fr (A.-F.M.); jehrhart@hotmail.fr (J.E.); cmingo@chimie.ups-tlse.fr (C.M.); souchard@chimie.ups-tlse.fr (J.-P.S.); poinso@chimie.ups-tlse.fr (A.P.); 2 Université de Toulouse, Equipe de Biophysique Cellulaire, IPBS-CNRS UMR5089 205, Route de Narbonne BP 64182, 31077 Toulouse, France; laure.gibot@ipbs.fr (L.G.); lucwasungu@yahoo.com (L.W.); rols@ipbs.fr (M.-P.R.); 3 Université de Toulouse, Laboratoire de Chimie Agro-industrielle (LCA), INRA, INPT, INP-EI PURPAN, 31076 Toulouse, France; frederic.violleau@purpan.fr

**Keywords:** polymer micelles, pheophorbide-a, cellular penetration, uptake mechanism

## Abstract

Drug delivery by nanovectors involves numerous processes, one of the most important being its release from the carrier. This point still remains unclear. The current work focuses on this point using poly(ethyleneglycol-b-ε-caprolactone) micelles containing either pheophorbide-a (Pheo-a) as a fluorescent probe and a phototoxic agent or fluorescent copolymers. This study showed that the cellular uptake and the phototoxicity of loaded Pheo-a are ten times higher than those of the free drug and revealed a very low cellular penetration of the fluorescence-labeled micelles. Neither loaded nor free Pheo-a displayed the same cellular localization as the labeled micelles. These results imply that the drug entered the cells without its carrier and probably without a disruption, as suggested by their stability in cell culture medium. These data allowed us to propose that Pheo-a directly migrates from the micelle to the cell without disruption of the vector. This mechanism will be discussed.

## 1. Introduction

Amphiphilic block copolymer micelles are nano-sized self-assembled particles with diameters between 10 and 100 nm [1,2]. These particles can increase the solubility of hydrophobic molecules due to their unique structural composition, which includes a hydrophobic core surrounded by a hydrophilic corona [3,4,5,6,7]. The former serves as a reservoir in which drugs can become incorporated through various interactions depending on their physicochemical properties [8]. The hydrophilic corona ensures colloidal stability and biodispersity of the micelle. These nanocarriers can be designed to escape opsonization and the mononuclear phagocytic system [9], which is one of the most important biological barriers to controlled drug delivery. This results in increased plasma half-life [10] and extended circulation time [11,12]. Further, these nanocarriers can benefit from the enhanced permeability and retention effect, resulting in drug accumulation in cancerous tissues [13,14]. Therefore, micelles are powerful and promising drug delivery systems that can improve drug solubility, pharmacokinetics, and biodistribution. Extensive studies have been performed on this subject, including innovative approaches for developing advanced and stimuli-responsive micelles for biomedical applications [15,16,17,18,19,20,21,22,23,24].

However, the biological processes involved in the cellular interactions and uptake of nanocarriers remain poorly understood. Due to the diversity of copolymer systems, there is no consensus on these points. Increased understanding of these processes is essential for improving the efficiency and innocuity of nanocarriers.

Kabanov’s group reported that Pluronic unimers and corresponding micelles are internalized by endocytosis through two different pathways [25]. Pluronic unimers primarily penetrate through caveolae-mediated endocytosis, whereas micelles internalize through clathrin-mediated endocytosis [26]. Savic et al. [12,27,28] developed several fluorescent poly(ethyleneoxide-b-ε-caprolactone) (PEO-PCL) copolymers that enabled visualization of interactions between micelles and cells by confocal microscopy. They were the first group to assess the issue of cellular penetration and subcellular localization of copolymer micelles [29]. Nanocarriers were reported to enter cells through endocytosis after non-specific interactions with the cell membrane [12,27,29,30]. In contrast to Pluronics, PEO-PCL micelles are frozen systems. Thus, the involvement of unimers in the drug delivery process might be minor, and the mechanism of penetration might be entirely different. In 2008, Chen et al. [31] proposed that [(polyethylene glycol)-b-poly (d,l-lactide)] copolymer micelles did not penetrate into cells but enabled increased transfer of an entrapped drug through the plasma membrane, resulting in cellular penetration of the drug. However, the mode of penetration remained unclear. These reports suggest that the modes of cellular penetration may differ for each type of copolymer. The effects of size and core/shell structure on the biological fate or cellular interactions of polymeric micelles also remain poorly characterized [32]. Drug delivery by polymeric micelles or nanoparticles is often determined by tracking a fluorophore that is initially encapsulated inside the carrier. Unfortunately, uncontrolled release of the fluorophore from nanoparticles may produce unreliable results. In addition, although the results of fluorophore tracking are generally used to describe the drug delivery process, the molecular structures of the drug and fluorophore are usually quite different. Thus, applying the results of fluorophore tracking experiments to drug delivery is questionable, because the delivery mechanism is highly dependent on the load [33]. Careful investigations into the biological processes mediating contact between polymeric micelles and cells or in vivo are of paramount importance for increasing knowledge of these macromolecular particles and developing future drug delivery systems.

The aim of this paper was to analyze the mechanism by which a micelle made of poly(ethylene oxide)-poly(ε-caprolactone) mediates drug delivery and to identify the multifactorial aspects of this issue. Pheophorbide-a (Pheo-a) was selected as a fluorescent probe and as a phototoxic hydrophobic drug.

## 2. Results and Discussion

### 2.1. Characterization of Copolymer Micelles

**Figure 1:**
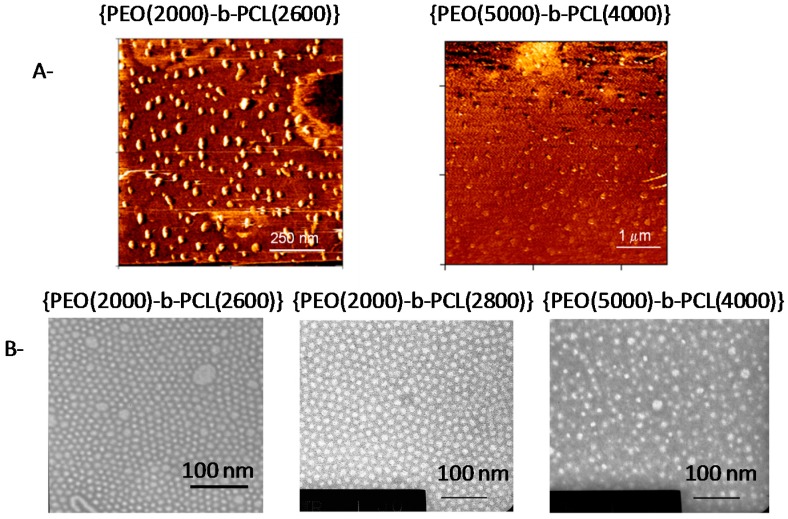
(**A**) AFM pictures of self-assembled nanoparticles. Left: {PEO(2000)-b-PCL(2600)}, right: {PEO(5000)-b-PCL(4000)}; (**B**) Typical TEM images of poly(ethylene oxide-b-ε-caprolactone) micelles. Left: {PEO(2000)-b-PCL(2600)}, center: {PEO(2000)-b-PCL(2800)}, right: {PEO(5000)-b-PCL(4000)}. The bar represents 100 nm in all TEM images.

{PEO(2000)-b-PCL(2600)}, {PEO(2000)-b-PCL(2800)}, and {PEO(5000)-b-PCL(4000)} micelles with or without encapsulated Pheo-a were prepared and characterized by DLS (Figures S2 and S3), TEM, AFM (Figure 1), Differential Scanning Calorimetry and asymmetrical flow field-flow fractionation (AsFlFFF) [34] (Table 1).

Nanoparticles have a diameter range between 18 nm and 27 nm in water. AFM and TEM (Figure 1) suggested that copolymers formed spherical nano-objects. All the methods yielded results that were consistent with nanoparticles having diameters around 20 nm. As expected, the average diameter determined by TEM was smaller than the hydrodynamical diameter measured in solution.

No strong variation in this diameter was recorded in comparison to the exact composition of the polymers. DLS also suggested that the hydrodynamical diameters of the particles were more or less constant for all of the polymers selected for this study. Dissolution of Pheo-a within micelles may induce mild swelling of nano-objects as suggested by comparing the diameters of objects with or without Pheo-a, 22 and 27 nm, respectively, for {PEO(2000)-b-PCL(2800)} and 25 and 26 nm, respectively, for {PEO(5000)-b-PCL(4000)}). These data demonstrate that nano-objects are preserved in the presence of Pheo-a.

The diameters extracted from the AsFlFFF experiments were quite similar regardless of the analytical method [34]. Characterization was completed by SANS for some systems in order to gain more insight into the organization of the core/shell (Table 2). The Guinier regime of the scattering curve indicated a homogeneous sphere with a diameter around 15 nm in the case of {PEO(2000)-b-PCL(2600)}. This was highly consistent with the size determined by DLS and AsFlFFF.

**Figure 2:**
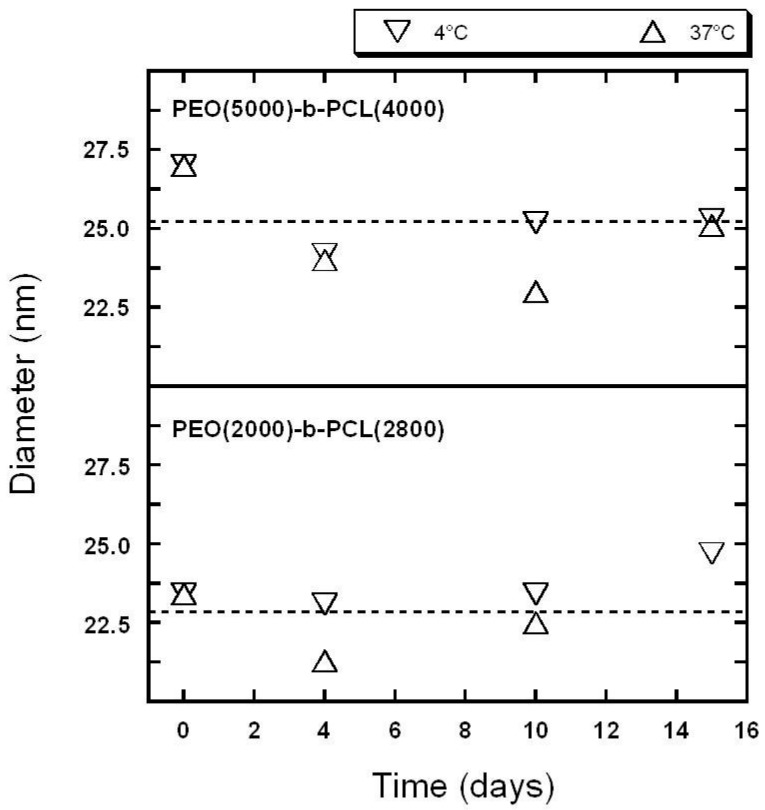
Evolution of self-assembly size over time estimated by DLS. {PEO(2000)-b-PCL(2800)} at 4 °C, {PEO(2000)-b-PCL(2800)} at 37 °C, {PEO(5000)-b-PCL(4000)} at 4 °C, {PEO(5000)-b-PCL(4000)} at 37 °C. Dashed line: average diameters for all of the data (temperature and times).

A model of hairy spheres (dense homogeneous spherical core on which polymers are grafted) fits the experimental curves and suggests a diameter of approximately 10 nm for the inner part of the particles and a 2.5-nm thick shell. SANS results also suggested that dissolution of Pheo-a within the micelles induced a very slight increase in the diameter of the nano-objects. The stabilities of polymeric micelles in solution were assessed in absence of proteins by DLS at 37 °C and 4 °C. The results for empty polymeric micelles are presented in Figure 2.

No significant differences were recorded between measurements at low and high temperatures. Intensity mean analysis showed that changes in size remained minor over a time period of more than 14 days. This was consistent with our published results [35].The loaded polymeric micelles exhibited the same trend (data not shown).

### 2.2. Cellular Uptake and Phototoxicity

**Scheme 1:**
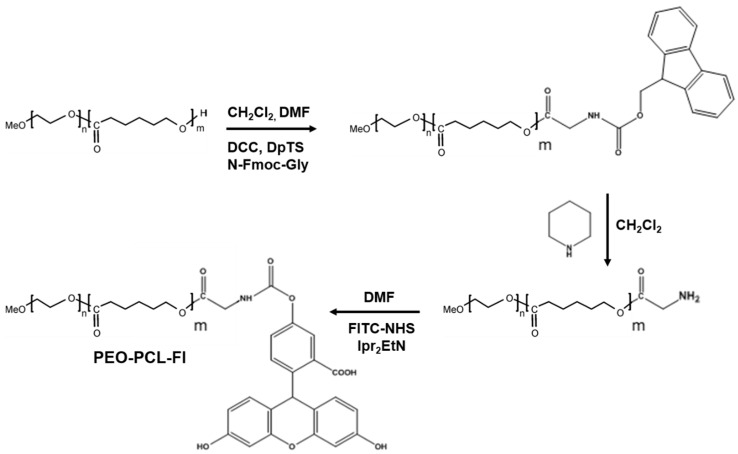
Synthesis of PEO-PCL Fluorescein.

Cell penetration studies were performed with HCT-116 human colon cancer cells with the copolymer {PEO(2000)-b-PCL(2600)} labeled (at 2 or 4 mol %) or not with fluorescein (Scheme 1). The labeled polymer, in which fluorescein was covalently linked to the PCL part of the copolymer, was characterized by ^1^H-NMR and size-exclusion chromatography (Figure S4). Incorporation of labeled polymers into unlabeled polymeric micelles was examined by AsFlFFF chromatography (Figure S5).

Pheo-a was encapsulated in copolymer micelles at different molar ratios (R) of Pheo-a to copolymer unimers of 1/50 and 1/30. The final concentration of Pheo-a in cell culture media was kept constant (10^−6^ M). The kinetics of cellular penetration of free or encapsulated Pheo-a and fluorescein-labeled copolymer micelles were determined at 37 °C and 4 °C.

**Figure 3:**
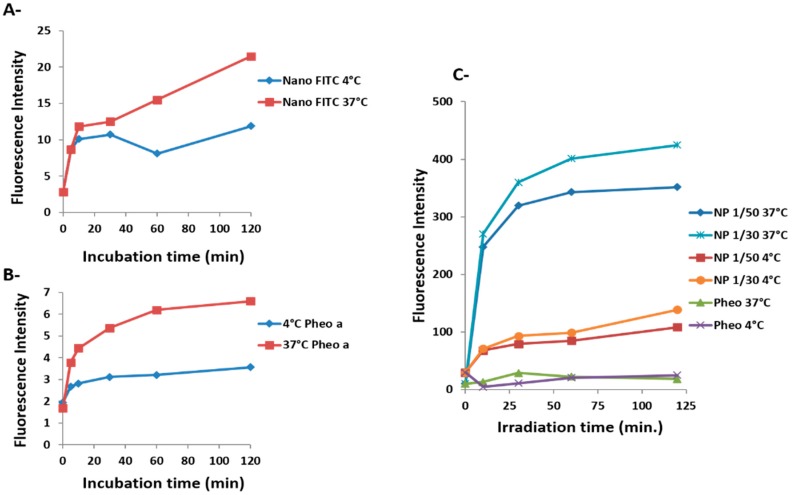
Evaluation of cellular uptake of Pheo-a by flow cytometry versus incubation time and temperature. (**A**) {PEO(2000)-b-PCL(2600)} copolymer micelles labeled with fluorescein; red line, *T* = 37 °C; blue line, *T* = 4 °C; (**B**) Free Pheo-a (10^−6^ M; red line, *T* = 37 °C; blue line, *T* = 4 °C; (**C**) Loaded Pheo-a in copolymer micelles at molar ratios of 1/50 and 1/30. Each set of experiments and experimental conditions were performed twice.

The mean fluorescence of Pheo-a or fluorescein in intact cells was assessed by flow cytometry. A very low cellular penetration was observed for copolymer micelles labeled with fluorescein (Figure 3A). This low uptake was attributed to a poor internalization of the micelles, since fluorescence quenching was not observed in a control experiment for this concentration of fluorescein.

Cellular entry was time-dependent, and equilibrium was not reached within 24 h of incubation. Therefore, this process showed small amplitude and was very slow. In contrast, internalization of encapsulated Pheo-a increased sharply (Figure 3C). This process was dependent on time and loading concentration and reached a plateau after 30 min of incubation. It exhibits two phases: a fast one, of high amplitude, and a slow one of small amplitude. The fluorescence of loaded Pheo-a micelles in cells at R 1/50 or 1/30 was 10 or 12 times higher than that of free Pheo-a, respectively (Figure 3B).

**Figure 4:**
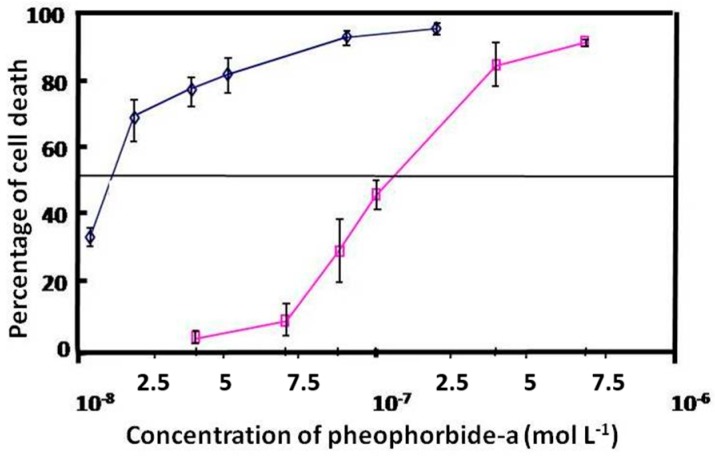
Phototoxicity of free Pheo-a (purple line) or Pheo-a encapsulated in {PEO(2000)-b-PCL(2800)} micelles (blue line). HCT-116 cells were incubated for three hours with various concentrations of Pheo-a and then irradiated. Cell viability was evaluated by MTT test reduction assay 48 h after PDT treatment. Results were expressed as mean ± S.D. of five replicates.

These data clearly show that copolymer micelles promote a significant increase in cellular uptake of Pheo-a. Photocytotoxicity study of free and encapsulated Pheo-a on cellular proliferation of HCT-116 cells showed a ten-fold enhancement of the PDT activity of Pheo-a when it was delivered by copolymers micelles (Figure 4) which is directly correlated to the increase of its cellular penetration.

These results suggest that cellular penetration of encapsulated Pheo-a in copolymer micelles involves at least two mechanisms. One mechanism is fast and does not seem to involve internalization of the micelles with Pheo-a but may correspond to cellular uptake of the major part of the encapsulated Pheo-a alone. The other mechanism is slow and may be related to cellular internalization of micelles with Pheo-a.

Endocytosis is often examined in studies involving mechanisms of micelle cellular entry. For this purpose we performed the same set of experiments at 4 °C to examine the mechanisms of micelle cellular penetration. This thermal condition produced a sharp decline in cellular penetration of encapsulated Pheo-a of around 70% (Figure 3C). Similarly, internalization of the free photosensitizer appeared to be partially inhibited (Figure 3B). These results suggest that endocytosis is not the only mechanism involved in the penetration.

### 2.3. Confocal Microscopy Investigations

**Figure 5:**
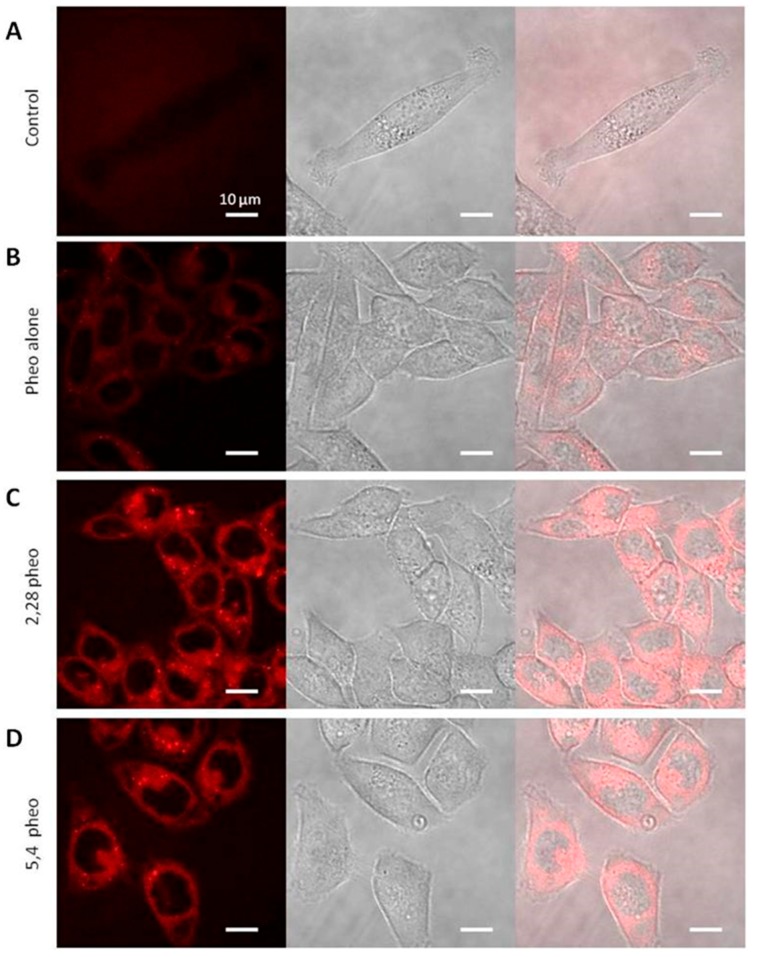
Cellular internalization of Pheo-a observed by confocal microscopy. PEO-PCL micelles loaded with Pheo-a at a ratio of 1/30. (**A**) Control of HCT-116 cells autofluorescence in the same excitation and emission conditions as those used for fluorescein (λ_excitation_: 633 nm, λ_emission_ > 640 nm); (**B**) HCT-116 cells incubated for 60 min in the presence of Pheo-a (10^−5^ M), λ_excitation_: 633 nm, λ_emission_ > 640 nm; (**C**) HCT-116 cells incubated for 80 min in the presence of Pheo-a (10^−6^ M) incorporated into {PEO(2000)-b-PCL(2800)}; (**D**) HCT-116 cells incubated for 80 min in the presence of Pheo-a (10^−6^ M) incorporated into {PEO(5000)-b-PCL(4000)}micelles. Images were obtained with a Zeiss LSM710 confocal microscope. G×60 (Marly le Roi, France).

**Figure 6:**
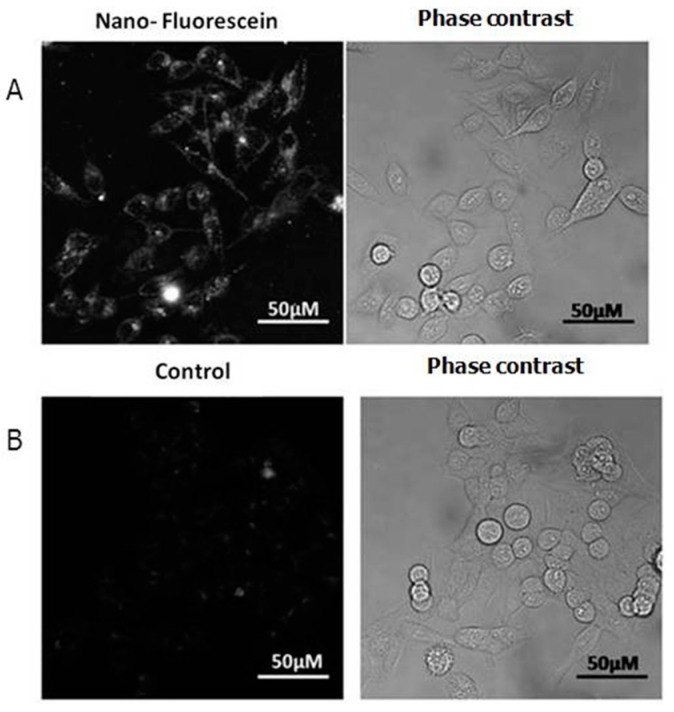
Cellular internalization of fluorescein-labeled PEO-PCL micelles observed by confocal microscopy. (**A**) HCT-116 cells incubated for 80 min in the presence of fluorescent polymer, λ_excitation_: 405/488 nm, λ_emission_: 488 nm; (**B**) Control of HCT-116 cell autofluorescence in the same excitation and emission conditions as those used for fluorescein. Images were obtained with a Zeiss LSM710 confocal microscope.

Confocal microscopy confirmed strong cellular penetration of Pheo-a encapsulated in {PEO(2000)-b-PCL(2800)} and {PEO(5000)-b-PCL(4000)} (Figure 5). Free (Figure 5B) and encapsulated (Figure 5C,D) Pheo-a have both the same cellular distribution. It is therefore important to note that copolymer micelles do not affect Pheo-a subcellular distribution. Copolymer micelles labeled with fluorescein showed low fluorescence in cells, confirming a poor cellular penetration (Figure 6). The cellular distribution was more punctiform and differed from that of the free or delivered Pheo-a. These results highlight an important discrepancy between the internalization and the sub-cellular localization of fluorescently labeled micelles and those of the encapsulated Pheo-a.

Taken together, the data strongly suggest that a high proportion of loaded Pheo-a in micelles does not enter into cells in the encapsulated form and might follow the same mechanism of cellular entry as that of free Pheo-a leading to a similar subcellular localisation.

This is consistent with published results on cellular penetration [12,27] and shows that copolymer micelles mediate entry of Pheo-a into cells. The data strongly suggest that the encapsulated dye is no longer associated with the micelle as it enters the cell and that the main process of its transfer to the cell does not involve endocytosis of micelles. Thus, free and micelle-loaded Pheo-a would be expected to follow the same cellular uptake pathway from the cell membrane resulting in similar subcellular distribution.

In a previous work [36], we have studied the stability of copolymer micelles in biological media and we have shown that they slowly dissociated in the presence of proteins and FBS. Therefore, dissociation of micelles does not occur before penetration of Pheo-a molecules, which typically occurred in less than 2 h in our in vitro experiments. PEO-PCL micelles are stable in cell culture media. Thus, it seems unlikely that dynamic instability of micelles contributes to release of Pheo-a in the vicinity of the cell membrane as was suggested for PEG-PDLLA micelles [31]. Transfer of Pheo-a from the core micelle to the cell membrane without release of the photosensitizer in cell culture media provides a possible mechanism.

Delivery of the hydrophobic drug does not only depend on micelle stability but is also dependent on the partition coefficient in the micelle/biological environment of the loaded molecule. Maysinger [37] and Lavasanifar [32] showed that incorporation of DiIC into a PEO-PCL micelle resulted in very poor internalization of the fluorescent probe in comparison to free DiIC. These data support our assumption of drug transfer. It is conceivable that the differential internalization of Pheo-a and incorporated DiIC micelle may be partly due to different degrees of partitioning between the polymer and cell membrane. Pheo-a is less hydrophobic than DiIC and may escape more easily from the micelle core to the cell membrane.

Consequently, the main transfer of Pheo-a from micelles to cells does not involve endocytosis of micelles and is not due to dissociation of the micelle. As proposed by Kerdous et al. a possible mechanisms is a direct transfer by direct collision between micelles and biological membranes (collision model) [38]. Moreover, Kerdous et al. provide strong evidence against copolymer micelle disruption and Pheo-a release into cell culture media. This transfer might be facilitated by the PEO corona by inducing dehydration of the lipid bilayer and enhancing membrane permeability [31]. These results indicate that the affinity of the drug for the micelle will determine the kinetics of exchange between the micelle core and cell membrane. This direct transfer appeared to be the major drug delivery process mechanism of PEO-PCL micelles in our experimental conditions. However, there remains the possibility that a small amount of drug may penetrate by endocytosis of micelles, as suggested by experiments at 4 °C which showed a sharp decrease in cellular uptake of encapsulated and free Pheo-a. Direct transfer of Pheo-a from the micelle to the membrane may also be slower at this temperature. Furthermore, although the glass transition temperature of PCL blocks has not been reached (−58 °C), a decrease in temperature from 37 °C to 4 °C would reduce drug mobility inside the polymer matrix. To the best of our knowledge, the only paper that presented a similar concept was a study on poly(lactide-co-glycolide) nanoparticles described by Xu et al. [39]. In that study, Nile Red was encapsulated in nanoparticles, and very rapid cellular penetration of Nile Red was observed. However, when the nanoparticle was labeled, fluorescence increased very slowly over a 24-h period. Therefore, the fluorophore penetrated without its cargo similar to our observations. Further analysis of the penetration kinetics of doxorubicin and paclitaxel suggested two potential mechanisms of drug release. Release of drug may be followed by transfer to the cell membrane, or direct transfer of drug from the carrier to the membrane may occur.

## 3. Materials and Methods

### 3.1. Chemicals and Materials

Poly(ethylene oxide-b-ε-caprolactone) {PEO(2000)-b-PCL(2600)}, {PEO(2000)-b-PCL(2800)}, and {PEO(5000)-b-PCL(4000)} were purchased from Gearing Scientific (Hertfordshire, SG7 5LL, UK). Purity and molar mass were checked by ^1^H-NMR on a Brucker AC-300 spectrometers (Wissembourg Cedex, France) and size-exclusion chromatography. The solvents (from SDS, Seihl, France) were used as received. Pheo-a was obtained from a published protocol [34] or from Wako Chemicals GmbH (Neuss, Germany). Pheo-a stock solutions (1 mM) were prepared in ethanol or acetone and stored at −18 °C. Experimental Pheo-a aqueous solutions were prepared from the stock solution, handled in the dark, and used extemporaneously. All other chemicals were purchased from Sigma-Aldrich (Saint-Quentin Fallavier, France).

### 3.2. Preparation of Polymer Micelles

Preparation was previously described [35]. Briefly, preparation of polymer micelles was performed by dispersing an acetone polymer solution into ultrapure filtered water. The solution was left standing for two days to eliminate acetone.

### 3.3. Dynamic Light Scattering (DLS)

DLS was carried out at 25 °C on a Malvern Zetasizer NanoZS (Malvern Instruments, Orsay Cedex, France). Solutions were analyzed as synthesized without filtration to ensure that undesired populations were not removed. Data were analyzed by the general-purpose non-negative least squares (NNLS) method. The typical accuracy for these measurements was 10%–15%.

### 3.4. Differential Scanning Calorimetry (DSC)

The influence of Pheo-a on the polymer morphology inside the polymeric self-assembly was assessed by DSC on a sensitive instrument (DSC1 equipped with an HSS8 sensor, Mettler Toledo, Viroflay, France). First, a 10 wt % solution of {PEO(2000)-b-PCL(2800)} in water was prepared by directly dispersing the polymer in water with sonication. The DSC analysis of this solution is reported in Figure S1A and exhibited the expected fusion of poly(ε-caprolactone) segments, which usually occurs at 57 °C for the bulk polymer. Four solutions of polymeric micelles were analyzed in the same manner at 0.4 wt %, which corresponds to the concentration during regular synthesis. Both {PEO(2000)-b-PCL(2800)} and {PEO(5000)-b-PCL(4000)} were used, and the incorporation of Pheo-a 1/30 mol/mol was assessed. The results (Figure S1B) show differences in crystallinity between the empty polymeric self-assemblies and the ones loaded with Pheo-a. Indeed, no fusion was observed in the presence of Pheo-a, corroborating encapsulation of Pheo-a inside micelles close to the poly(ε-caprolactone) core. Comparison of the areas of the peaks also indicated that poly(ε-caprolactone) chains had a lower crystallinity ratio in the polymeric micelles compared to those of the rough dispersions. The fusion enthalpy was evaluated at 59.5 J·g^−1^ for the rough dispersion compared to 16.2 and 7.7, respectively, for {PEO(2000)-b-PCL(2800)} and {PEO(5000)-b-PCL(4000)} self-assemblies.

### 3.5. Atomic Force Microscopy (AFM)

Samples were spread on glass slides and analyzed in contact mode in dry conditions on a NanoWizard^®^ II Life Science Version from JPK Instruments (Berlin, Germany). Some slides were pretreated with oxygen plasma to avoid dragging of micelles with the AFM tip.

### 3.6. Small Angle Neutron Scattering (SANS)

SANS experiments were performed with the PACE spectrometer at the Orphée reactor (Laboratoire Léon Brillouin, Saclay, France). Polymer-D_2_O mixtures (polymer concentration 4 mg·mL^−1^) were placed inside quartz cells with a gap of 2 mm at 20 °C. Data were collected in three spectrometer configurations, which were determined by the neutron wavelength and the sample-to-detector distance (12 Å, 4.7 m), (5 Å, 4.7 m), and (5 Å, 1.3 m). The total wave-vector (q) range covered 0.002–0.30 Å^−1^. Scattered intensities were corrected for empty-cell scattering. Detector normalization was performed by dividing data by the scattering from 1 mm of light water. Data were converted to an absolute scale (I(q) = d∑/δΩ, cross-section per unit volume in cm^−1^) with instrument constants provided by LLB and subtracting the incoherent background.

### 3.7. Transmission Electron Microscopy (TEM)

TEM experiments were performed with a HU12A microscope (Hitachi, Velizy Cedex, France, accelerating voltage of 75 kV). Small amounts of particle suspensions in water were deposited onto a discharged copper grid coated with a carbon membrane and wiped with absorbing paper. A few drops of uranyl acetate solution were deposited onto the grid for 30 s, and the grid was then dried under a lamp for three minutes.

### 3.8. The Asymmetric Flow Field Flow Fractionation

(AsFlFFF) instrument was an Eclipse 2 System (Wyatt Technology Europe, Dernbach, Germany). The AsFlFFF channel had a trapezoidal geometry with a length of 17.3 cm, an initial breadth of 1.1 cm, and a final breadth of 0.27 cm. A 250-µm thick Mylar spacer was placed between the ultrafiltration membrane and the upper glass plate. The accumulation wall was an ultrafiltration membrane of regenerated cellulose with a 10-kDa cut-off (Wyatt Technology Europe). An Agilent 1100 Series Isocratic Pump (Agilent Technologies, Waldbronn, Germany) with an in-line vacuum degasser and an Agilent 1100 Autosampler delivered the carrier flow and handled sample injection into the AsFlFFF channel. A 0.1-µm in-line filter (VVLP, Millipore, Darmstadt, Germany) was installed between the pump and the AsFlFFF channel. The products were detected with 18 angles Multi-Angle Light Scattering (MALS) DAWN-DSP (Wyatt Technology, Santa Barbara, CA, USA), an OptilaRex Refractometer (Wyatt Technology), and an Agilent 1100 UV detector (λ = 214 nm or 412 nm). The MALS detectors were normalized with bovine serum albumin. Calibration of scattering intensity was performed with HPLC-grade filtered toluene. Water, which was filtered with 0.02% sodium azide before use, (vacuum filtration system using Gelman filters of 0.1 µm) was used as an eluent. For separation, the channel flow rate was fixed at 1 mL·min^−1^, and the cross-flow rate varied. For empty nanoparticles, the separation program started with 4 min with a 1 mL·min^−1^ cross-flow rate. For micelles containing Pheo-a, the separation program started with 2 min with a 0.2 mL·min^−1^ and 2 min with a 1.5 mL·min^−1^ cross-flow rate. The system then switched to Focus mode, and the cross-flow rate was stabilized 1 min before injection at 1 mL·min^−1^. Five microliters of the sample solution were injected into the AsFlFFF channel at a flow rate of 0.2 mL·min^−1^ for two min. After injection, one minute of focus was maintained before the elution started.

In elution mode and for empty nanoparticles, the cross-flow rate was fixed at 1 mL·min^−1^ for 10 min. Cross flow was then stopped in order to eliminate all particles present in the AsFlFFF system. For micelles containing Pheo-a, the cross-flow rate was primarily maintained for 2 min at 0.5 mL·min^−1^. After the cross-flow rate decreased linearly for two minutes, elution followed at 0.2 mL·min^−1^ for 20 min. The flow-rate through the detectors, *V_out_*, was constantly maintained at 1 mL·min^−1^. The refractive index increment dn/dc of the copolymer micelles in water was measured with a PSS dndc-2010 instrument at 620 nm and was determined to be 0.125 mL·g^−1^.

### 3.9. Synthesis of PEO-PCL-Fluorescein

4-(Dimethylamino)pyridinium 4-toluenesulfonate (DpTS) was synthesized as follows (Scheme 1): monohydrated *p*-toluenesulfonic acid (1.5 g) (*M* = 190.22 g·mol^−1^, 7.9 mmol) was dissolved in 40 mL toluene. The solution was distilled with Dean Stark glassware to eliminate traces of water. Heating was discontinued when the level of toluene decreased by 75%, and the solution was cooled to room temperature. Dimethylaminopyridine (0.96 g) (*M* = 122.17 g·mol^−1^, 7.9 mmol, 1.0 eq) in 20 mL toluene was added slowly under an argon atmosphere. The precipitate of DpTS was filtered and recrystallized in 1,2-dichloroethane.

#### 3.9.1. Synthesis of PEO-PCL-Fmoc

Three equivalents of fmoc-Gly-OH (*M* = 297.31 g·mol^−1^) was dissolved in dry *N*,*N*-dimethylformamide (DMF) (1 g of fmoc-Gly-Oh in 4 mL DMF). One equivalent of DpTS and six equivalents of *N*,*N*′-Dicyclohexylcarbodiimide (DCC) (*M* = 206.33 g·mol^−1^) were added to this solution under argon (Scheme 1). The solution was stirred at room temperature for two hours (h), producing a white precipitate. A 10^−2^ M solution of the polymer in dichloromethane (CH_2_Cl_2_) (1 eq) was added and stirred for a further 72-h period. The precipitate was filtered, and the remaining solution was evaporated. The resulting solid was dissolved in a minimal amount of CH_2_Cl_2_, and ethanol was added (final EtOH/CH_2_Cl_2_ 4/1). Methylene chloride was evaporated, and the solution was stored in the freezer for one night. The resulting white solid was filtered and washed with small quantities of ethanol (EtOH). ^1^H-NMR (CDCl_3_, displacement shifts in ppm vs. TMS): 1.3 (m, 45H, -CH_2_- caprolactone), 1.55 (m, 90H, -CH_2_- caprolactone), 2.2 (t, 45H, -CH_2_-CO caprolactone), 3.26 (s, 3H, OMe), 3.55 (bs, 165H, -CH_2_- PEO), 3.8 (t, 1H, fmoc), 3.85 (d, 2H, fmoc), 3.95 (t, 45H, -CH_2_-O- caprolactone), 4.05 (t, 1H, fmoc), 4.3 (d, 2H, fmoc), 7.2 (t, 2H, fmoc), 7.25 (t, 2H, fmoc), 7.5 (d, 2H, fmoc), 7.65 (d, 2H, fmoc).

#### 3.9.2. Synthesis of PEO-PCL-NH_2_

PEOPCL-fmoc (1 g) was dissolved in 15 mL CH_2_Cl_2_, and 86 mg of piperidine (0.1 mL, *M* = 85.15 g·mol^−1^, 5 eq) was added under argon (Scheme 1). The solution was stirred for 48 h, and the solvent was then evaporated. The remaining product was dissolved in a minimal amount of CH_2_Cl_2_, and ethanol was added (final EtOH/CH_2_Cl_2_ 4/1). Methylene chloride was evaporated, and the solution was stored in the freezer for one night. The resulting white solid was filtered and washed with small quantities of EtOH. ^1^H-NMR (CDCl_3_, displacement shifts in ppm vs. TMS): 1.35 (m, 45H, -CH_2_- caprolactone), 1.6 (m, 90H, -CH_2_- caprolactone), 2.25 (t, 45H, -CH_2_-CO caprolactone), 3.3 (s, 3H, OMe), 3.55 (bs, 165H, -CH_2_- PEO), 4.05 (t, 2H, -CH_2_-NH_2_), 4.1 (t, 45H, -CH_2_-O caprolactone).

#### 3.9.3. PEO-PCL-Fl

PEO-PCL-NH_2_ (1 g) was dissolved in 20 mL dry DMF. Ten equivalents of diisopropylethylamine was added under argon followed by three equivalents of fluorescein-*N*-hydroxysuccinimide (*M* = 473.4 g·mol^−1^) (Scheme 1). The solution was stirred at room temperature for 48 h. The solvent was evaporated, and the remaining product was dissolved in the minimum amount of CH_2_Cl_2_. Ethanol was then added (final EtOH/CH_2_Cl_2_ 4/1). Methylene chloride was evaporated, and the solution was stored in the freezer for one night. The resulting white solid was filtered and washed with small quantities of EtOH. PEO-PCL-FI was obtained with a yield of 80%. ^1^H-NMR (acetone-*d*_6_, displacement shifts in ppm vs. TMS): 1.4 (m, 45H, -CH_2_- caprolactone), 1.6 (m, 90H, -CH_2_- caprolactone), 2.3 (t, 45H, -CH_2_-CO caprolactone), 3.26 (s, 3H, OMe), 3.55 (bs, 165H, -CH_2_- PEO), 4.1 (t, 45H, -CH_2_-O caprolactone), 6.5–6.8 (m, 6H, fluorescein), 7.3 (m, 1H, fluorescein), 7.9 (m, 1H, fluorescein), 9.0 (bs, 2H, fluorescein).

### 3.10. Cell Line and Cell Culture

The HCT-116 cell line (ATCC #CCL-247) originates from a human colorectal carcinoma. Cells were grown in Dulbecco’s Modified Eagle’s Medium (DMEM; Invitrogen, Carlsbad, CA, USA) containing 4.5 g/L glucose, L-glutamine, and pyruvate and supplemented with 10% (*v*/*v*) heat-inactivated fetal calf serum, 100 U/mL penicillin, and 100 µg/mL streptomycin. Cells were maintained at 37 °C in a humidified atmosphere containing 5% CO_2_ (Incubator Jouan, St. Herblain, France).

### 3.11. Measure of Uptake by Flow Cytometry

HCT-116 cells were seeded at 50,000 cells per well in 24-well plates one day before the experiment. Cells were incubated with 500 µL media containing either free Pheo-a or Pheo-a-loaded nanoparticles. The final concentration of Pheo-a was 1 µM. After gentle trypsinization, the Pheo-a cellular content was quantified by flow cytometry (FACSCalibur, Becton Dickinson, San Jose, CA, USA).

### 3.12. Cellular Imaging

HCT-116 cells were seeded on Lab-Tek chamber cover glass (Nunc, Illkirch, France) overnight at 37 °C in a humidified atmosphere with 5% CO_2_. Cells were incubated at 37 °C for 60 min with 10 µM of either {PEO(2000)-b-PCL(2800)} or PEO(5000)-b-PCL(4000)}Pheo-a loaded nanoparticles. Cell imaging was performed with a FLUOVIEW FV1000 confocal laser-scanning microscope (Olympus France, Rungis, France).

### 3.13. Phototoxicity

HCT-116 cells were seeded into 12-well plates at a concentration of 2 × 10^4^ per well. After 24 h, increasing amounts of Pheo-a were added to cell culture solubilized in DMSO and diluted 10^3^-fold directly into culture media or incorporated into {PEO(2000)-b-PCL(2800)}copolymer micelles suspended in water and diluted 10^2^-fold into media. After 3-h incubation, cells were washed with PBS, and fresh culture media was added. Illumination was performed with an overhead projector with a band-pass filter (λ > 400 nm, 8.2 J·cm^−2^). The plates were placed back into the incubator and continually incubated for 2 days. Fresh media was provided every day. The 3-(4,5-dimethylthiazole-2-yl)-2,5-biphenyltetrazolium bromide (MTT) reduction assay, which has been widely used to measure the relative PDT effects of dyes, was used to monitor the photocytotoxicity of free Pheo-a or Pheo-a incorporated into nanoparticles. The mean values and the standard deviations between measurements were calculated from five independent experiments (three wells per concentration). Phototoxic responses were fitted with a sigmoid dose–response curve.

## 4. Conclusions

Only a few teams have examined the mechanisms of action involved in the drug release from copolymer micelles. These studies are the source of many debates that underscore the complexity of the mechanisms involved. This study demonstrates that PEO-PCL micelles facilitate high delivery of Pheo-a to cells. The major mechanism of Pheo-a drug delivery by this type of micelle was direct transfer of the hydrophobic drug from the micelle to the cell membrane. This mechanism occurred without disruption of the micelle or drug release in the vicinity of the cell membrane. In these conditions, micelles did not modify the subcellular distribution of the hydrophobic drug. Our results and those of the literature [9,27,28,29,30,31,38,39,40,41,42,43,44,45,46] suggest that different mechanisms of drug delivery are possible depending on the drug/polymer couple: (i) low penetration of the drug and carrier; (ii) drug release followed by transfer to the cell membrane; (iii) direct transfer of the drug upon contact of the carrier with the cell membrane. This behavior will be critically linked to the partition coefficient of the drug and its affinity towards possible media, i.e., polymer carrier, extracellular media, or cell membrane. This study highlights the great care that should be taken when considering the fundamental description of drug delivery by polymeric micelles or other carriers. For each system, it is essential to examine the whole delivery system, which includes the carrier, drug, and biological environment.

## Figures and Tables

**Table 1:** Characterization of PEO-PCL self-assemblies in water.

Polymer	Average Dh (Batch DLS) (nm) ^a^	Average D (TEM) (nm)	Average Dh (DLS AF4) (nm)	Nagg ^b^
{PEO(2000)-b-PCL(2600)}	17.7	13.7 ± 2.7	20.2	200
{PEO(2000)-b-PCL(2800)}	22.0	13.7 ± 2.7	22.4	N.D
{PEO(2000)-b-PCL(2800)} + Pheo 1/30	27.2	16.8 ± 2.7	27.4	N.D. ^c^
{PEO(5000)-b-PCL(4000)}	24.6	12.6 ± 2.7	26.8	190
{PEO(5000)-b-PCL(4000)} + Pheo 1/30	25.6	N.D	N.D	N.D.

^a^ The estimated accuracy with which all of the experiments can be reproduced is 15%. The range values provided for TEM data correspond to the nanoparticle polydispersity; ^b^ determined from $\bar{M_{w\;agg}}/\bar{M_{w\;polymer}}$; ^c^ not determined due to cross-interference of Pheo with light scattering calculation.

**Table 2:** SANS characterization of PEO-PCL micelles.

Sample	Guinier			Hairysphere Model		
	R_g_	R(_sphere_)_eq_	Nagg (R_sphere_)	R_core_	Nagg (R_core_)	Total Diameter
{PEO(2000)-b-PCL(2600)}	5.6 nm	7.3 nm	396–222	5.0 nm	127	14.8 nm
{PEO(2000)-b-PCL(2600)} + Pheo 1/10	6.4 nm	8.3 nm	582–326	5.2 nm	143	15.2 nm
{PEO(5000)-b-PCL(4000)} + Pheo 1/20	7.1 nm	9.2 nm	515–227	6.0 nm	143	ca. 12 nm

## Supplementary Materials

Supplementary materials can be accessed at: http://www.mdpi.com/1420-3049/21/12/1643/s1.
