# Supplementary material for: Drug Release by Direct Jump from Poly(ethylene-glycol-b-ε-caprolactone) Nano-Vector to Cell Membrane
Source: Molecules. 2016 Nov 30;21(12):1643. doi: 10.3390/molecules21121643 (PMC6273951; doi:10.3390/molecules21121643)
Supplement: Supplementary file 1 [file molecules-21-01643-s001.pdf]

Supplementary Materials: Drug Release by Direct Jump from Poly(ethyleneglycol-b-ε-caprolactone) Nano-Vector to Cell Membrane

Ugo Till, Laure Gibot, Anne-Françoise Mingotaud, Jérôme Ehrhart, Luc Wasungu, Christophe Mingotaud, Jean-Pierre Souchard, Alix Poinso, Marie-Pierre Rols, Frédéric Violleau and Patricia Vicendo

A

B

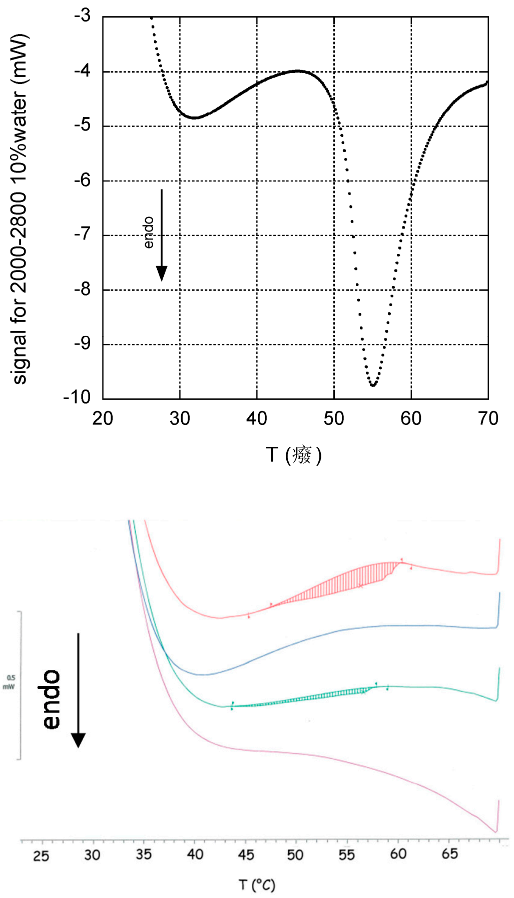

Figure S1. (**A**) DSC analysis of a 10% wt solution of {PEO(2000)-b-PCL(2800)} in water; (**B**) DSC analysis of 0.4% wt polymeric self-assemblies in water. Red upper curve: 2000-2800, blue curve: 2000-2800 1/30 Pheo-a, green curve: 5000-4000, purple lower curve: 5000-4000 1/30 with Pheo-a.

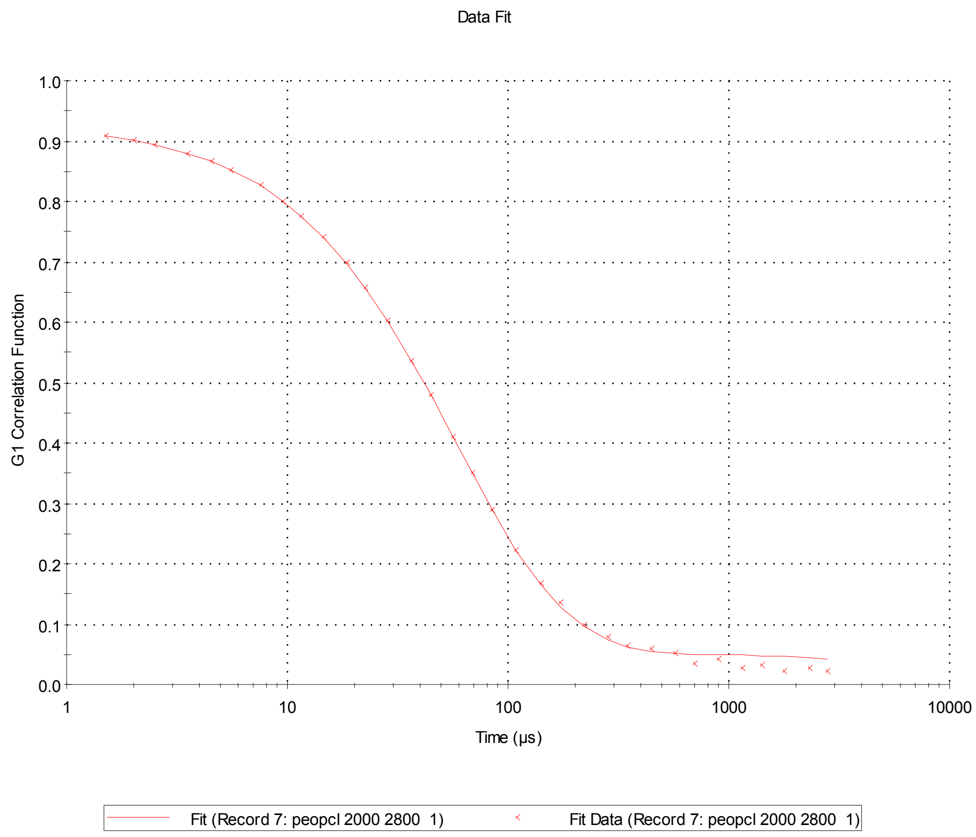

Figure S2. Typical correlogram and fit for DLS analyses.

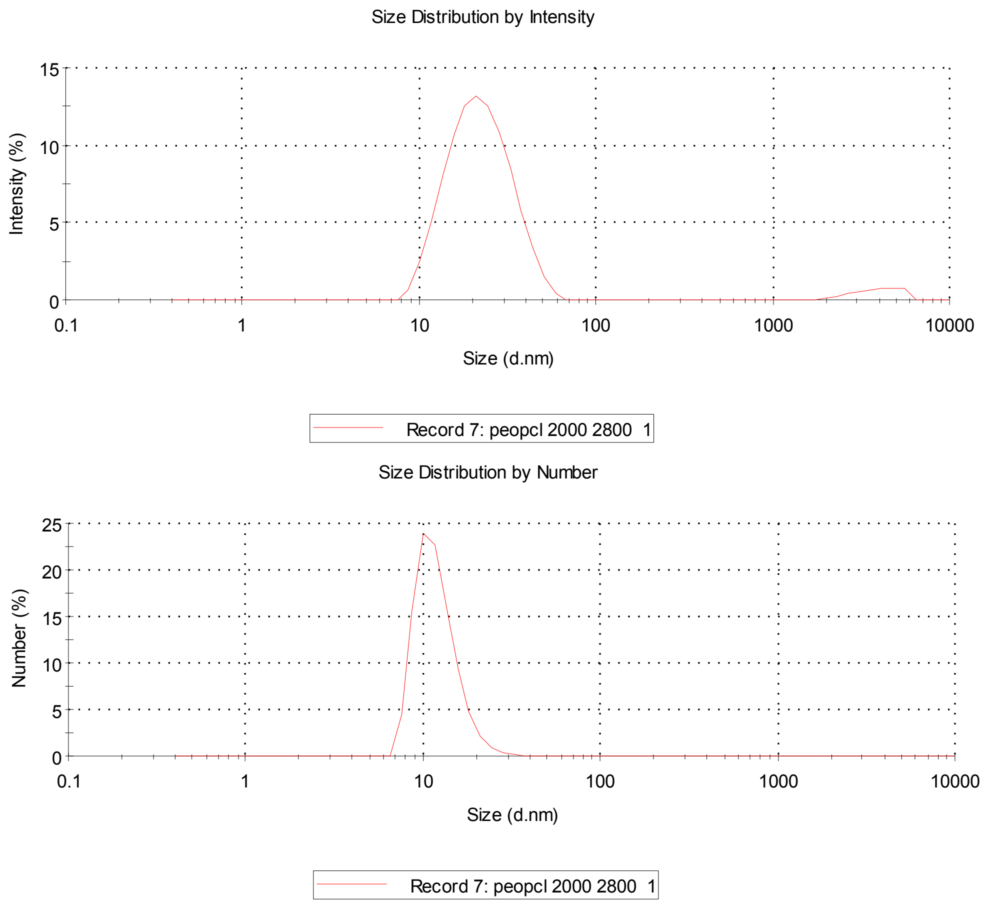

Figure S3. Typical DLS analysis by intensity or number means.

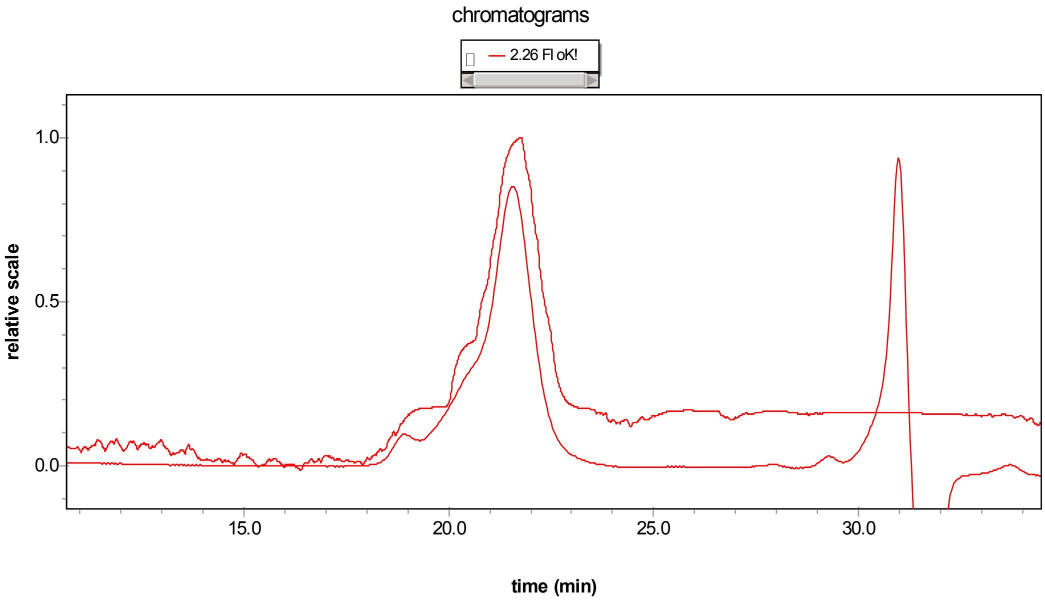

Figure S4. Size-Exclusion Chromatography of PEO-PCL-fluorescein. Full line, UV signal; dotted line, refractometric signal. The right-sided peak on the refractometric signal was due to solvent injection.

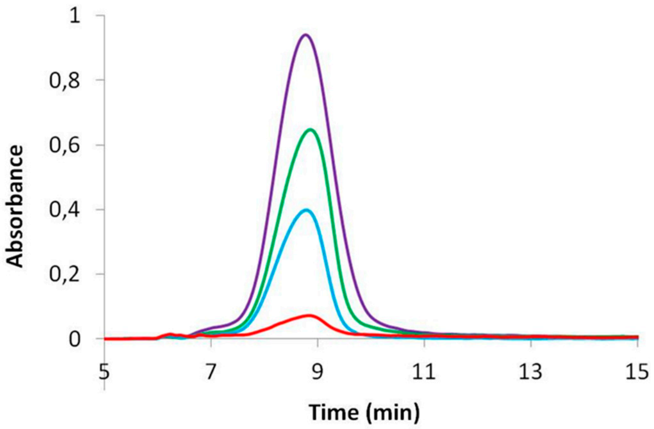

Figure S5. AFlFFF analysis of {PEO(2000)-b-PCL(2600)} micelles with increased ratios of PEO-PCL-fluorescein. Red 0%, blue 2 mol %, green 4%, purple.
